# Prolonged-release opioid agonist therapy: qualitative study exploring patients’ views of 1-week, 1-month, and 6-month buprenorphine formulations

**DOI:** 10.1186/s12954-019-0296-4

**Published:** 2019-04-03

**Authors:** Joanne Neale, Charlotte N. E. Tompkins, John Strang

**Affiliations:** 1 National Addiction Centre, Institute of Psychiatry, Psychology & Neuroscience, King’s College London, 4 Windsor Walk, London, SE5 8AF UK; 2 South London and Maudsley NHS Foundation Trust, Maudsley Hospital, Denmark Hill, London, SE5 8AZ UK

**Keywords:** Prolonged-release buprenorphine, Opioid agonist therapy, Extended-release OAT, Methadone, Buprenorphine, Depot injections, Implants, Medication duration, Qualitative study

## Abstract

**Background:**

Options for opioid agonist therapy (OAT) are expanding with the development of prolonged-release (also known as extended-release) 1-week, 1-month, and 6-month formulations of buprenorphine. There is an assumption that patients will welcome these new treatments and medication adherence will correspondingly increase. However, there has been little research exploring patients’ views of prolonged-release buprenorphine. This paper aims to understand which durations patients prefer and why, and to consider the findings with reference to the development of future OAT products.

**Methods:**

Data were generated as part of a qualitative interview study. Fieldwork was conducted in London, UK, during 2018 (before any prolonged-release OAT formulations were licensed in Europe). Participants (*n* = 36) were taking daily oral OAT (methadone or buprenorphine) or using heroin daily without OAT. They included 26 men and 10 women, aged 24–63 years. All were asked for their views on weekly, monthly, and six-monthly duration buprenorphine. Responses were audio-recorded, transcribed, and analyzed by Iterative Categorization.

**Results:**

Participants generally stated that having buprenorphine of different prolonged durations was positive. They tended to believe that ‘longer’ prolonged-release formulations would be beneficial for patients who wanted to avoid thinking about drugs and drug-using associates, wished to evade the stigma of substance use, and desired ‘normality’ and ‘recovery.’ In contrast, participants favored ‘shorter’ prolonged-release formulations for patients who are new to OAT, worried about the safety and reliability/effectiveness of OAT, want a ‘break’ from street opioids, and need contact with services to monitor/support them. Participants indicated that transitioning between OAT medications of different duration would be a very individual process. Some also linked prolonged-release OAT duration to political, philosophical, and ethical issues, such as patient coercion and mental capability.

**Conclusions:**

Medication duration is an important but complex feature of prolonged-release buprenorphine, with patients’ views and preferences likely to be influenced by a wide range of factors. We need further qualitative research to explore the experiences of people who have actually used prolonged-release OAT. Meanwhile, drug developers should continue to build flexibility and choice into OAT products to ensure that future treatment is acceptable to patients and able to accommodate their diverse individual needs.

## Background

Opioid agonist therapy (OAT) is a well-evidenced treatment for opioid use disorder that involves the prescription of pharmaceutical opioids such as the agonist (methadone) and the partial agonist (buprenorphine) [1, 2]. Historically, OAT medications have nearly always been prescribed for daily consumption in liquid/linctus or tablet/film form. In recent years, however, treatment options have started to expand with the development and approval of a range of prolonged-release (also known as extended-release) weekly, monthly, and six-monthly buprenorphine products (see Table 1). In Europe, the first prolonged-release products (weekly and monthly buprenorphine depot injections) were licensed for use in November 2018 [3].

**Table 1:** Licensed prolonged-release opioid agonist therapy products at 31st December 2018

Pharmaceutical drug	Delivery system	Duration	Brand name	Company	Countries where licensed (year of approval)
Buprenorphine	Depot injection	1 month	Sublocade™	Indivior, Richmond, VA, USA	USA (2017)
Buprenorphine	Depot injection	1 week and 1 month	Buvidal®/ CAM2038	Camurus AB, Lund, Sweden	Europe (2018) and Australia (2018)
Buprenorphine	Implant	6 months	Probuphine®	Titan Pharmaceuticals, Inc., San Francisco, CA, USA	USA (2016)

As a partial agonist, buprenorphine produces less respiratory depression and weaker feelings of intoxication than full agonists (such as heroin, diamorphine, or methadone) [4]. At low doses, it prevents withdrawal symptoms and the desire to use illicit opioids. Meanwhile, high doses do not increase intoxication [4, 5]. Buprenorphine does not cause the cycle of highs and lows linked to opioid misuse and is consequently associated with a lower risk of abuse, side effects, and overdose than full opioid agonists. Despite this, it can precipitate withdrawal symptoms if taken by a person with an opioid dependence who has a full agonist in their bloodstream [2, 4–6].

Recent investment in prolonged-release buprenorphine (implant and depot injection formulations) has been prompted by global increases in opioid use and poor adherence to existing short-acting opioid medications [7–9]. Patients treated with implants and depot injections do not need to collect their medication daily from services; a change which is expected to reduce the treatment burden for clinicians and patients, prevent medication diversion, increase treatment compliance, and benefit patients living in areas where access to medical facilities or clinics is limited [6–8]. Nonetheless, there is a lack of research evidence supporting these assumptions. This is because studies of new medicinal products typically focus on product characterization, safety, and efficacy, without considering how patients might use, not use, or misuse medications once they are approved and being prescribed within real world settings [10–14].

Responding to this information gap, we previously undertook a focus group study (6 focus groups, 44 participants) with current and former OAT patients who all had a history of heroin use in order to explore their views of different routes of OAT medication administration [14, 15]. This research found that participants expressed both positive and negative opinions of implants and depot injections. For example, they generally welcomed being free from having to constantly attend services and appointments—emphasizing that this would reduce the stigma they often felt as OAT patients, enable them to do other things, and help them begin to live ‘normal’ lives. Despite this, they worried about medication administration, effectiveness, side effects, overdosing, lack of dose control, and stopping treatment once they had started. Some additionally felt that they might be socially isolated without their daily visit to the pharmacy or would miss the daily ‘highs’ and ‘lows’ associated with oral methadone [14, 15].

Our analyses led us to conclude that prolonged-release implant and depot formulations of OAT are not ‘fixed’ medications that can be administered to people to achieve pre-determined treatment outcomes [14]. Instead, they are more usefully viewed as complex ‘assemblages’ generating uncertain outcomes. For example, an implant or depot injection comprises a pharmaceutical drug or drugs (e.g., buprenorphine), a dose, with a duration of action, fitted in a certain way, and manufactured to disperse medication in a particular manner. Additionally, implants and depot injections are themselves part of wider interacting ‘assemblages’. Thus, they might generate side effects, interact with other medications, be better understood via information sources, and be evaluated by their impact on feelings of control, daily rituals and habits, relationships, ability to travel, or employment opportunities [14].

In this paper, we build on our prior work by taking an in-depth look at one specific feature of prolonged-release OAT: medication duration. To this end, we conducted a new qualitative interview study to explore potential patients’ views and expectations of the three buprenorphine dosing options that are currently available: 1 week, 1 month, and 6 months. This topic seems particularly important to consider given that these time periods are each associated with very different reductions in service contact compared with daily dosing formulations. In undertaking this analysis, our aim is to better understand which medication durations patients prefer and why, but also to consider our findings with reference to the development of future OAT products.

## Methods

The opportunity to conduct this work arose when one of the companies developing prolonged-release buprenorphine formulations was responsive to our request for research funding to explore prolonged-release depot buprenorphine, and willingness to receive prolonged-release depot buprenorphine, from the perspective of people who use opioids. We conducted our qualitative interview study in London, UK, between June and October 2018 (before any prolonged-release OAT formulations had been licensed for use in Europe). Ethical approval was granted by a university research ethics committee. People were eligible to participate if they were over 18 years of age and taking prescribed daily oral methadone, or prescribed daily oral buprenorphine, or using heroin daily but not taking any OAT. These criteria were chosen to ensure that the study participants were broadly reflective of people who could potentially be eligible for prolonged-release OAT, yet also included people who were using opioids but might feel that OAT was not appropriate for them.

In total, 36 people were interviewed. These included 26 men and 10 women, aged 24–63 years (mean 45 years). Most (*n* = 24) identified as White British. Twelve were taking prescribed oral methadone, 12 were taking prescribed oral buprenorphine, and 12 were using heroin daily but not currently prescribed any OAT. Just over a third (*n* = 16) were collecting OAT medication from a pharmacy daily, five were collecting OAT medication 2–3 times a week, and three were collecting OAT medication weekly. Over half (*n* = 21) reported that they were currently using heroin (see Table 2 for participant characteristics).

**Table 2:** Participant characteristics

	Total (*n* = 36)
Sex	
Male	26 (72%)
Female	10 (28%)
Age (years)	
Mean (range)	45 (24–63)
Ethnicity	
White/White British	24 (67%)
Black/Black British	5 (14%)
Asian/Asian British	1 (3%)
Mixed or multiple	3 (8%)
Other	3 (8%)
Current prescribed medication	
Daily oral methadone	12 (33%)
Daily oral buprenorphine	12 (33%)
Daily heroin (not in treatment)	12 (33%)
Frequency of medication collection from a pharmacy	
Daily	16 (44%)
2–3 times a week	5 (14%)
Weekly	3 (8%)
Not applicable	12 (33%)
Current heroin use	
Yes	21 (58%)
No	15 (42%)

Participants were recruited using similar procedures from two alcohol and other drug (AOD) treatment services, two homeless hostels, and an AOD peer support service. To begin, the study researcher (CT) briefed service staff on the study aims and methods. Next, service staff approached people whom they considered to be eligible, provided them with verbal information about the study, and asked them if they were willing for their contact details to be forwarded to the research team. CT then used the information received to contact all interested people, describe the study further, confirm eligibility, and select people with a mix of demographic characteristics and current medication experiences to participate in a semi-structured interview at a time that was convenient to them.

Participants were all given a written information sheet and asked to sign a consent form before their interview started. The information sheet clearly stated that the study was funded by a pharmaceutical company developing prolonged-release depot buprenorphine. However, CT emphasized that the research team was independent from the company. Using a topic guide, CT then explored participants’ personal circumstances, substance use, treatment history, and views on prolonged-release depot buprenorphine. To facilitate the discussions, she also gave all participants basic verbal information on key features of a product concept of a prolonged-release buprenorphine depot injection (summarized in Table 3). If participants expressed interest, CT additionally showed them a prototype depot injection prefilled syringe device that contained no active medication. The prototype was placed on a table in a clear zip-sealed bag for participants to view.

**Table 3:** Summary of the key features of the prolonged-release buprenorphine depot injection product concept presented to participants

Overview: provided for the treatment of opioid dependence.	
Effectiveness: designed to help reduce withdrawal, craving, and patients’ use of illicit opioids.	
Duration: available as weekly and monthly depot injections.	
Dosage: designed to deliver a set dose every day; medication dose can be ‘topped up’ if needed; dose reductions are only possible at the end of the week/month; there is the option to move between weekly and monthly injections.	
Administration: under the skin (subcutaneous) injection into the patient’s arm, buttock, stomach, or thigh by a healthcare professional.	
Potential side effects: comparable to daily sublingual buprenorphine, except for mild to moderate injection site reactions (e.g., pain, itching, red skin, swelling, lump around the injection site).	
Service attendance: no need for supervised daily dosing; may only need to attend a clinic or pharmacy on the day of the injection, either once a week or once a month.	

About half way through the interviews, participants were asked for their views on medication duration. To this end, they were invited to comment on why they thought people might or might not want a weekly buprenorphine depot medication, why they might or might not want monthly buprenorphine depot medication, why they might want or might not want a hypothetical buprenorphine depot medication that lasted 6 months, which duration they would personally prefer, and why they thought people might want to change between medication durations. The hypothetical 6-month depot medication was discussed rather than a 6-month implant in order to keep participants focused on the duration of the medication and not distract them by introducing a different delivery system.

Interviews were all conducted in private in rooms at the five participating services and were audio recorded. They lasted 37–100 min (median 67 min). On completion of their interview, each participant was given a £20 shopping voucher. Audio recordings were transcribed verbatim and the transcriptions were entered into the software programme MaxQDA v11 for coding [16]. To this end, CT developed a coding frame based on deductive codes derived from the topic guide and inductive codes emerging from the interview data. She next coded all the interview data line-by-line to the coding frame. For this paper, all data coded to the medication duration codes were exported into Word documents for analyses following the stages of Iterative Categorization (as outlined below) [17].

First, CT worked through all the Word documents and summarized the coded data descriptively, keeping a close record of which participants made which points. These summaries were then passed to JN, who reviewed them more interpretively to identify patterns and themes in the data. Next, JN re-organized all the analyses under four inductive headings: (i) duration choice and preferences, (ii) reasons and situations for favoring ‘longer’ prolonged-release formulations, (iii) reasons and situations for favoring ‘shorter’ prolonged-release formulations, and (iv) reasons for transitioning between durations. At this point, the analyses were returned to CT for further review, team discussion, and finalization. Findings are presented below and illustrated using anonymized verbatim quotations.

## Results

### Duration choice and preferences

Whilst a few participants expressed confusion regarding the reason why different durations had been developed, most felt that having medications of different durations was positive since this gave patients choice and meant that treatment could be better tailored to meet their individual needs:

I suppose you will probably get some people that would prefer the 7-day one, other people 28 days: different strokes for different folks. (Participant 19, buprenorphine, male)

Of the three medication durations, the 6-month version generated the most divergent views. Thus, some stated that they would ‘love it’ and it would be ‘amazing’ and the ‘best drug ever’. In contrast, others expressed very negative views. For example, some argued that nobody should be maintained on OAT for 6 months as the goal must always be reduction leading to abstinence; some voiced concerns that a 6-month formulation might release too much medication at once, so causing people to overdose, or have other long-term harmful side effects; and some expressed disbelief that a 6-month depot injection could ever be effective:

I wouldn’t believe it… I don’t think there is such a drug that can last for six months. (Participant 7, heroin, female)

Overall, however, participants did not tend to express a definitive preference for one duration over another; rather they identified reasons why a ‘longer’ or ‘shorter’ duration formulation would be better in specific situations or circumstances or for achieving particular treatment-related goals.

### Reasons and situations for favoring ‘longer’ prolonged-release formulations

Participants consistently reported that a depot injection that lasted a month, and ideally 6 months, would be more valuable than a weekly depot injection in terms of increasing their ability to live a ‘normal life.’ A key explanation that participants gave for this was that people using opioids would not need to attend treatment services or pharmacies so often, and this would result in them having more time and opportunity to do other more ‘normal’ things, such as travel, work, go on holiday, visit friends, and be spontaneous:

[If] you’ve got something that’s going to last 28 days, you can go on holiday, you can go and visit someone. (Participant 18, buprenorphine, male)

Several participants also stated that a medication which could stop cravings or withdrawal symptoms for weeks or months would mean that people no longer needed to commit crimes to acquire money for drugs. Over time, participants believed that the ability to live a more ‘normal’ life would help patients feel more positive about themselves:

I’d be able to get up and go to work like any normal person because… after a couple of months the thought goes in my head, like I’m not even a drug addict anymore. (Participant 32, heroin, male)

Relatedly, many participants explained how the longer (1-month or 6-month) depot formulations would remove the stigma and embarrassment they routinely experienced as a result of having to take their medication in a community pharmacy in front of other customers. They also said that the longer formulations would free them from ‘bumping into’ people who tried to sell or give them drugs in and around pharmacies and treatment services. Equally, they explained that the longer formulations would alleviate the stress and inconvenience they often experienced from having to arrange their days around attending appointments and pharmacies:

People haven’t got to worry about going to the chemist daily. They haven’t got to worry about being back at a certain time. And so, like if they did get a job and they had to work over the time the chemist’s shutting, they wouldn’t have to worry about, ‘Oh I’ve got to leave work’ or ‘going to have to tell my manager’. (Participant 28, methadone, female)

Participants sometimes added that having a medication that lasted a month or longer would provide them with welcome respite from the physical symptoms of withdrawal (being ‘sick’) and the constant psychological worry that they might become ‘sick.’ In turn, they said that this would have an important further benefit of reducing the time that they spent thinking about obtaining and using drugs:

[In] 28 days you can forget about everything about it [heroin]… Four weeks instead of thinking [about it] every day. (Participant 15, buprenorphine, male)

For these various reasons, participants often reported that the 1-month and 6-month depot injections were likely to be more appropriate for people who were ‘serious’ about ‘staying away from’ heroin than the weekly depot injection. Some participants also noted that the 6-month depot injection would offer people more time to really begin to address other issues underlying their substance use, change their thinking about drugs, modify their drug-taking behaviors and rituals, and lay the foundations of ‘recovery’:

You’ve got six months to sort of like get your head round it, work with it and recover. (Participant 18, buprenorphine, male)

In contrast, participants stated that the weekly formulation would not give people time to make any fundamental life changes before the medication ended, so making it relatively easy for people to go back to using street drugs.

Finally, several participants suggested that longer formulations would be beneficial in prisons. For example, they said that they would reduce illicit substance use, medication diversion, and prison violence, and this would ultimately save the prison service resources (even though they acknowledged that some prisoners might not like prolonged-release OAT). In addition, one participant reported that a 6-month depot injection could have a useful role as an ‘enforced’ community treatment—providing a better alternative to a prison sentence for people who broke the law:

If they were going out thieving, then I believe six months [depot injection] straightaway… possibly enforced by a judge if they’re in court… I think that would be preferable to sending someone to jail for 12 months. (Participant 26, methadone, male)

### Reasons and situations for favoring ‘shorter’ prolonged-release formulations

Participants (particularly those currently taking prescribed methadone and those using heroin but not in treatment) repeatedly argued that the weekly depot injection was best for initiating treatment. Specifically, they reported that the shorter, weekly duration would enable people to ‘try the medication out’ before committing to it, adding that 7 days is ‘less daunting’ when people do not know what to expect:

I would start off with once a week to see how I feel… to see how I go with it, because obviously it’s new. See how your body works with it. (Participant 5, methadone, female)

Having a shorter initial treatment period would also, they said, enable doctors to check that the patient was committed to treatment, and give patients an opportunity to be sure that they felt comfortable with the dose they had been given and confident that the medication would work for them. Indeed, participants often suggested that the 1-week depot injection would be better for people who were apprehensive or worried about OAT, concerned that depot medication might have negative short- or long-term side effects, or anxious that longer formulations might not last as long as the drug developers promised:

A month is like stressful getting up every day, thinking is it going to wear off today? (Participant 16, buprenorphine, female)

As participants further explained, people who received the weekly depot injection would have less time to wait for the medication to finish if they did not like the treatment or if it ‘disagreed with them’. Equally, those who were less committed to being in treatment would be able to use heroin again after only a week (participants noted that they could stop their treatment completely or simply take a break from treatment and use heroin for a period before having another buprenorphine injection):

You can have a break off the drugs for seven days, and when that’s over you can go on a seven-day mad one, have a bit of heroin. (Participant 12, heroin, male)

Additionally, some participants (particularly those already being prescribed methadone and buprenorphine daily) suggested that the shorter weekly and monthly depot injections would be better than a longer 6-month option for people who were detoxing or who wanted to be abstinent since medication doses could be tapered down more quickly:

If it’s seven days… it would be quicker for you to get there for the lower dose. Otherwise you’ve got to wait the whole of the month before you can change it. (Participant 22, methadone, female)

Participants also argued that the weekly depot injection had the advantage of enabling people to maintain more regular contact with professionals and treatment services. A few commented that this was particularly important for people who might struggle to remember when their next depot injection was due. More often participants explained that a medication cannot work in isolation and so people need services to give them structure and support, to monitor their well-being, and to identify and react to any difficulties they are experiencing before these escalate:

They might be on a monthly one [depot] and they’re not… being seen [by professionals] often enough… They’ve hit a depression spot or something like that and they’re starting to try and use drugs again. You’ve got to try and catch them… before it goes wrong, so you can help them out. (Participant 11, methadone, male)

Lastly, one participant argued that a 6-month depot injection was problematic as people cannot think that far ahead and so are not capable of committing to being in OAT for that length of time.

### Reasons for transitioning between durations

Most participants recognized that it was good to have the option of transitioning between durations in case their circumstances altered or they changed their minds about their treatment. Furthermore, they identified specific reasons why patients might want to transition from shorter to longer and longer to shorter formulations. As previously indicated, participants often argued that it would be important to start with a weekly depot injection and then ‘work up’ to a monthly and subsequently a 6-month formulation as they became more used to, and comfortable with, the medication:

I would stick to the weekly one before I’d go to the monthly one. And then if everything was OK, then I would try the monthly one. Say after you tried the monthly one, they could come up with a 6-monthly one, say, and then you’d only have to go once a year, twice a year. That would be excellent. (Participant 3, heroin, male)

Indeed, one participant explained that each increase in duration would feel like a personal achievement, ‘demonstrating’ that people were making progress. Nonetheless, participants also often emphasized that once they were stable and doing well, they would want to transition back to shorter injection durations and lower medication dosages as part of a reduction plan that would ultimately allow them to achieve abstinence:

When you’ve reached your level and you’re happy and that and you’ve decided it’s time to come off, you can start going back to the weekly, lowering the dose. (Participant 17, buprenorphine, male)

Despite this, a few participants maintained that they would personally prefer to taper down their doses and detoxify using a 6-month formulation as the longer time period would be more comfortable and give them time to prepare mentally for being abstinent. This difference of opinion was captured in participants’ assertions that transitioning between depots of different duration would be a very personal process as ‘everyone is different.’

## Discussion

Although prolonged-release buprenorphine products are now being developed and approved internationally, there has been very little qualitative research exploring patients’ views and expectations of these new drug formulations. As we have previously argued, depot injections and implants are complex phenomena that comprise, inter alia, different pharmaceutical drugs, doses, durations, and ways of dispersing medication [14]. It is therefore important to research which features of these new products patients like, dislike, and worry about so that we can better understand why they might favor one product over another, why they might refuse or not comply with a given product, why their views and preferences might change over time, and how future formulations can be developed so they best meet patients’ needs. This information will also be key to the production of best practice guidelines and/or decision tools that will help clinicians and patients make informed choices when confronted with an increasing array of treatment options [for a further discussion see also 15].

In this paper, we focused on medication duration by limiting our discussion to one pharmaceutical drug (buprenorphine) and one delivery system (depot injection). Thus, we did not refer to implants and, for the purposes of these analyses, we did not consider dose. Despite this, participants still introduced variability into the interviews because they had their own personal experiences and understandings of OAT. For example, those currently taking daily prescribed buprenorphine appeared less likely to report that the weekly depot injection was good for initiating prolonged-release OAT (possibly because they were already used to, and comfortable with, with buprenorphine). Meanwhile, participants currently using heroin and not in treatment seemed less likely to suggest that shorter durations were better for detoxing and abstinence (possibly because ceasing OAT was less relevant to them than to participants currently taking prescribed methadone or buprenorphine).

The analyses presented are, in other words, limited because we could not completely isolate medication duration from other aspects of the prolonged-release OAT ‘assemblage’ [14]. Additionally, we were asking participants to discuss forms of prolonged-release OAT not available in the UK at the time of the interviews. What participants think about, and expect from, a treatment they have never actually had before may be very different from what they might report after they have personally experienced it. A further constraint on our analyses is that the data derive from a qualitative study involving 36 people from just 1 UK city. Consequently, we cannot draw any empirical generalizations or make any assumptions about how the findings would translate to other countries. Further, the sample size was too small to reliably identify any complex sub-group differences based on variables such as gender, race, or prior OAT experiences.

Despite these limitations, aspects of our findings are consistent with previous research into patients’ views of prolonged-release medication for opioid use disorder [14, 15, 18, 19] and this supports their trustworthiness [20]. Thus, analyses demonstrate that prolonged-release buprenorphine is a very complex treatment and participants’ views of medication duration were influenced by myriad factors. These included their desire to avoid being tied to services and appointments, experience less stigma, and do more ‘normal’ things; their concerns about treatment effectiveness, side effects, and overdosing; and their worries about having less professional support, losing the intoxicating effects of agonist drugs, and wanting to change their medication dose [14, 15, 18, 19]. Participants also generally appreciated why different durations could be useful and valued having choice and scope to move between the options. Nonetheless, a small number of participants did not immediately see why different durations existed, indicating that potential patients might benefit from information explaining the pros and cons of each.

Crucially, we found that longer duration formulations might be more suitable for patients who want to avoid thinking about drugs and other people who are using or dealing drugs, patients who are keen to evade the stigma of substance use, and patients who are seeking time and space to enjoy some ‘normality’ and establish routines and structure that will underpin more long-term ‘recovery.’ In contrast, shorter duration formulations might be better for patients who are new to OAT, are concerned about the safety and reliability/effectiveness of OAT, are interested in having a ‘break’ from using street opioids without necessarily stopping altogether, and need contact with services to monitor, provide structure, and support them. Although participants mostly thought that patients would find it easier to reduce and detoxify by transitioning from longer to shorter duration depot injections, a few participants reported that tapering their doses down using a 6-month prolonged-release formulation would offer an easier detoxification process.

Lastly, our findings suggest some political, philosophical, and ethical issues about prolonged-release OAT that merit consideration. First, participants did not tend to perceive it as a ‘maintenance’ medication; instead, they generally assumed that the endpoint would be detoxification and abstinence. Indeed, some participants questioned whether anyone should ‘ever’ be maintained on OAT. In addition, several participants suggested that prolonged-release OAT could be a useful criminal justice treatment, and one participant argued that people who use opioids are incapable of deciding to have a treatment that would last 6 months. Participants’ focus on abstinence (rather than maintenance) may have been influenced by the current emphasis on ‘recovery’ in UK policy and practice [21–23]; therefore, this finding might not be replicated in other countries and policy contexts. Coerced treatment, patient choice, and mental capability in treatment decision-making are, meanwhile, sensitive issues that have been debated in relation to other long-acting medications such as contraception [24, 25] and antipsychotic medications [26, 27]. It is difficult to draw any firm conclusions from the findings we report here, but the relationship between medication duration, choice, mental capability, and human rights will almost certainly feature in future discussions about prolonged-release OAT.

## Conclusions

The data presented indicate that medication duration is an important but complex feature of prolonged-release buprenorphine [14]. By analyzing the views and expectations of potential patients, we have been able to understand the issue of duration better. However, we now need further qualitative research to explore the experiences of people who have actually used prolonged-release OAT. This will enable us to more fully appreciate what patients like/dislike and are willing to adhere to in terms of duration but also in terms of other central features of prolonged-release products, such as the pharmaceutical drug, dose, delivery system, and dispersal mechanism. In the meantime, we recommend that drug developers continue to build as much flexibility and choice as possible into OAT products. This currently seems the best way of ensuring that tomorrow’s OAT will be acceptable to patients and able to accommodate their changing individual needs without undermining their freedom or human rights.

## Abbreviation

OAT: Opioid agonist therapy
